# On the inconsistency of *ℓ*_1_-penalised sparse precision matrix estimation

**DOI:** 10.1186/s12859-016-1309-x

**Published:** 2016-12-13

**Authors:** Otte Heinävaara, Janne Leppä-aho, Jukka Corander, Antti Honkela

**Affiliations:** 1 Helsinki Institute for Information Technology HIIT, Department of Computer Science, University of Helsinki, Helsinki, Finland; 2 Helsinki Institute for Information Technology HIIT, Department of Mathematics and Statistics, University of Helsinki, Helsinki, Finland; 3 Department of Biostatistics, University of Oslo, Oslo, Norway

**Keywords:** Gaussian graphical model, Structure learning, Inconsistency, Graphical lasso

## Abstract

**Background:**

Various *ℓ*_1_-penalised estimation methods such as graphical lasso and CLIME are widely used for sparse precision matrix estimation and learning of undirected network structure from data. Many of these methods have been shown to be consistent under various quantitative assumptions about the underlying true covariance matrix. Intuitively, these conditions are related to situations where the penalty term will dominate the optimisation.

**Results:**

We explore the consistency of *ℓ*_1_-based methods for a class of bipartite graphs motivated by the structure of models commonly used for gene regulatory networks. We show that all *ℓ*_1_-based methods fail dramatically for models with nearly linear dependencies between the variables. We also study the consistency on models derived from real gene expression data and note that the assumptions needed for consistency never hold even for modest sized gene networks and *ℓ*_1_-based methods also become unreliable in practice for larger networks.

**Conclusions:**

Our results demonstrate that *ℓ*_1_-penalised undirected network structure learning methods are unable to reliably learn many sparse bipartite graph structures, which arise often in gene expression data. Users of such methods should be aware of the consistency criteria of the methods and check if they are likely to be met in their application of interest.

## Background

Networks are ubiquitous in biology and inference of network structure from observed data is a common learning task. Many important biological networks have specific structural properties affecting this task. Gene regulatory networks, for instance, are nearly bipartite graphs with a small set of transcription factors regulating all the other genes. This structure has been successfully incorporated in gene regulatory network inference, often assuming a linear dependence between the regulators and targets, in both static (e.g. [1, 2]) as well as dynamic (e.g. [3, 4]) models. These fundamental assumptions form the basis for even very recent successful network inference projects (e.g. [5]).

The simplest and possibly the most widely used generic approaches for network inference are based on estimating the sparse precision matrix, i.e. the inverse covariance matrix, from data. The motivation for the approach stems from the fact that for a Gaussian Markov random field model, zeros in the precision matrix translate exactly to absent edges in the corresponding undirected Gaussian graphical model, thus being informative about the marginal and conditional independence relationships among the variables.

The full *p*-dimensional covariance matrix contains *p*(*p*+1)/2 parameters, making its accurate estimation from limited data difficult. Additionally, the structure learning requires the inverse of the covariance, and matrix inversion is in general a very fragile operation. To make the problem tractable, some form of regularisation is typically needed. Direct optimisation of the sparse structure would easily lead to very difficult combinatorial optimisation problems. To avoid these computational difficulties, several convex *ℓ*_1_-penalty-based approaches have been proposed. Popular examples include *ℓ*_1_-penalised maximum likelihood estimation [6], which also forms the basis for the highly popular graphical lasso (glasso) algorithm [7]. *ℓ*_1_ regularisation has also been used for example in a non-probabilistic alternative with linear-programming-based constrained *ℓ*_1_ minimisation (CLIME) algorithm [8].

At the heart of the optimisation problems considered by all these methods is a term depending on the *ℓ*_1_ norm of the estimated precision matrix. *ℓ*_1_-penalisation-based approaches such as lasso are popular for sparse regression, but they have a known weakness: in addition to promoting sparsity they also push true non-zero elements toward zero [9]. In the context of precision matrix estimation this effect would be expected to be especially strong when some elements of the precision matrix are large, which happens for scaled covariance matrices when the covariance matrix becomes ill-conditioned. This phenomenon occurs frequently under the circumstances where some of the variables are nearly linearly dependent.

In this paper we demonstrate a drastic failure of the *ℓ*_1_-penalised sparse covariance estimation methods for a class of models that have a bipartite structure where some variables depend linearly on others, such as in the commonly used and very successful gene regulatory network models. For such models even in the limit of infinite data, popular *ℓ*_1_-penalised methods cannot yield results that are significantly better than based on random guessing on any setting of the regularisation parameter. Yet these models have a very clear sparse structure that becomes obvious from the empirical precision matrix with an increasing *n*. Motivated by our discovery, we also explore the inconsistency of *ℓ*_1_-penalised methods on models derived from real gene expression data and find the methods poorly suited for such applications.

### Structure learning of Gaussian graphical models

We start with a quick recap on the basics of Gaussian graphical models in order to formulate the problem of structure learning. For a more comprehensive treatment of the subject, we refer to [10, 11]. Let **X**=(*X*_1_,…,*X*_*p*_)^*T*^ denote a random vector following a multivariate normal distribution with zero mean and a covariance matrix ***Σ***,**X**∼*N*_*p*_(**0**,***Σ***). Let *G*=(*V,E*) be an undirected graph, where the *V*={1,…,*p*} is the set of nodes and *E*⊂*V*×*V* stands for the set of edges. The nodes in the graph represent the random variables in the vector **X** and absences of the edges in the graph correspond conditional independence assertions between these variables. More in detail, we have that (*i,j*)∉*E* and (*j,i*)∉*E* if and only if *X*_*i*_ is conditionally independent of *X*_*j*_ given the remaining variables in **X**.

In the multivariate normal setting, there is a one-to-one correspondence between the missing edges in the graph and the off-diagonal zeros of the precision matrix ***Ω***=***Σ*** ^−1^, that is, *ω*_*ij*_=0⇔*X*_*i*_ ⊥ ⊥*X*_*j*_ |**X**∖{*X*_*i*_,*X*_*j*_} (see, for instance, [11], p. 129). Given an undirected graph *G*, a Gaussian graphical model is defined as the collection of multivariate normal distributions for **X** satisfying the conditional independence assertions implied by the graph *G*.

Assume we have a complete (no missing observations) i.i.d. sample **x**=(**x**_1_,…,*x*_*n*_) from the distribution *N*_*p*_(**0**,**Σ**). Based on the sample **x**, our goal in structure learning is to find the graph *G*, or equivalently, learn the zero-pattern of **Ω**. The usual assumption is that the underlying graph is sparse. A naive estimate for **Ω** by inverting the sample covariance matrix is practically never truly sparse for any real data. Furthermore, if *n*<*p* the sample covariance matrix is rank-deficient and thus not even invertible.

One common approach to overcome these problems is to impose an additional *ℓ*_1_-penalty on the elements of ***Ω*** when estimating it. This kind of regularisation effectively forces some of the elements of ***Ω*** to zero, thus resulting in sparse solutions. In the context of regression models, this method applied on the regression coefficients goes by the name of *lasso* [12]. There exists a wide variety of methods making use of *ℓ*_1_-regularisation in the setting of Gaussian graphical model structure learning [6–8, 13–16].

## Methods

### *ℓ*_1_-regularised methods for Gaussian graphical model structure learning

In this section we provide a brief review of selected examples of different types of *ℓ*_1_-penalised methods.

#### Glasso

We begin with the widely used graphical lasso-algorithm (glasso) [7]. Glasso-method maximises an objective function consisting of the Gaussian log-likelihood and an *ℓ*_1_ penalty:

1 $$ \log\det(\mathbf{\Omega}) - \text{trace}(\mathbf{\Omega S}) - \lambda |\mathbf{\Omega}|_{1},  $$

where **S** denotes the sample covariance matrix and *λ*>0 is the regularisation parameter controlling the sparsity of the solution. The *ℓ*_1_ penalty, $|\boldsymbol {\Omega }|_{1}= \sum _{i,j}|\omega _{ij}|$, is applied on all the elements of ***Ω***, but the variant where the diagonal elements are omitted is also common. (We use the notation |·|_*p*_ for the vector norm over matrix elements to avoid confusion with the matrix norm ∥·∥). The objective function (1) is maximised over all positive definite matrices **Ω** and the optimisation is carried out in practice using block-wise coordinate descent.

#### CLIME

The CLIME method (Constrained *ℓ*_1_ minimisation for Inverse Matrix Estimation) [8] approaches the problem of sparse precision matrix estimation from a slightly different perspective. It seeks matrices ***Ω*** with a minimal *ℓ*_1_ norm under the constraint

2 $$ |\mathbf{S}\boldsymbol{\Omega} - \mathbf{I}|_{\infty} \leq \lambda,  $$

where *λ* is the tuning parameter and |**A**|_*∞*_= max*i,j*|*a*_*ij*_| is the element-wise maximum. The optimisation problem min***Ω***|***Ω***|_1_ subject to the constraint (2) does not explicitly force the solution to be symmetric, which is resolved by picking from estimated values *ω*_*ij*_ and *ω*_*ji*_ the one with a smaller magnitude into the final solution. In practice, the optimisation problem is decomposed over variables into *p* sub-problems which are then efficiently solved using linear programming.

#### SCIO

The recently introduced Sparse Column-wise Inverse Operator (SCIO) [17] method decomposes the estimation of ***Ω*** into the following smaller problems

$$\min_{\boldsymbol{\beta}_{i} \in \mathbb{R}^{p}} \left\{ \frac{1}{2} \boldsymbol{\beta}_{i}^{T} \mathbf{S} \boldsymbol{\beta}_{i} - \mathbf{e}_{i}^{T} \boldsymbol{\beta}_{i} + \lambda |\boldsymbol{\beta}_{i} |_{1} \right\}, $$

where **S** and *λ* are defined as before and **e**_*i*_ is an *i*:th standard unit vector. The regularisation parameter *λ* can in general vary with *i* but this is omitted in our notation. The solutions $\hat {\boldsymbol {\beta }}_{i}$ form the columns for the estimate of ***Ω***. SCIO does not guarantee the symmetry of the resulting precision matrix, which is resolved as in the case of CLIME.

### Alternative methods

#### The naive approach

In addition to the above-mentioned *ℓ*_1_-penalised methods, we consider two alternative approaches. In a “naive” approach, we simply take the sample covariance matrix, invert it, and then threshold the resulting matrix to obtain a sparse estimate for the precision matrix. The threshold value is chosen using the ground truth graph so that the naive estimator will have as many non-zero entries as there are edges in the true graph. Setting the threshold value according to the ground truth is of course unrealistic, however, it is nevertheless interesting to compare the accuracy of this simple procedure to the performance of the more refined *ℓ*_1_ methods, when also their tuning parameters are chosen in a similar fashion.

#### FMPL

Lastly, we consider an approximate Bayesian approach which is based on finding a graph with the highest fractional marginal pseudo-likelihood (FMPL) [18]. Seeking the graph that maximises the marginal likelihood is equivalent with finding the maximum a posteriori graph, assuming a uniform prior over different graphs. However, computing the marginal likelihood is computationally challenging for a general graph, even in the Gaussian setting with conjugate priors. The FMPL method aims at circumventing this problem by replacing the true likelihood in the marginal likelihood with pseudo-likelihood. This leads to a convenient factorisation of marginal likelihood over variables and the resulting expression can be evaluated in closed form using previous results regarding objective comparison of Gaussian directed acyclic graphs [19, 20]. In practice, the factorisation allows the method to identify optimal Markov blankets independently for each of the variables using a greedy hill-climbing algorithm. The found Markov blankets are then combined into a proper undirected graph using any of the three different schemes commonly employed in graphical model learning: OR, AND and greedy hill-climbing (HC) [21].

### Model selection consistency

The assumptions required for a consistent model selection with an *ℓ*_1_-penalised Gaussian log-likelihood have been studied, for instance, in [22]. The authors provide a number of conditions in the multivariate normal model that are sufficient for the recovery of the zero pattern of the true precision matrix ***Ω*** ^∗^ with a high probability when the sample size is large. For our purposes, the most relevant condition is the following:

#### **Assumption 1**

There exists *α*∈(0,1], such that

3 $$  \gamma := \|\Gamma_{S^{C}S}{(\Gamma_{SS})}^{-1}\|_{\infty} \leq 1 - \alpha.  $$

Here *S*⊂*V*×*V* is a set defining the support of ***Ω*** ^∗^, that is, the non-zero elements of ***Ω*** ^∗^ (diagonal and the elements corresponding to the edges in the graphical model) and *S* ^*C*^ refers to the complement of *S* in *V*×*V*. The *Γ* term is defined via Kronecker product ⊗ as $\Gamma = (\boldsymbol {\Omega }^{*})^{-1} \otimes (\boldsymbol {\Omega }^{*})^{-1}\in \mathbb {R}^{p^{2}\times p^{2}}$ and *Γ*_*AB*_ refers to the specific rows and columns of *Γ* indexed by *A*⊂*V*×*V* and *B*⊂*V*×*V*, respectively. The norm in the equation is defined as $\|A\|_{\infty } = \max _{j} \sum _{i} |a_{ij}|$.

The above result applies to glasso. However, a quite similar result was presented for SCIO in [17]:

#### **Assumption 2**

There exists *α*∈(0,1), such that

$$\max_{1 \leq i \leq p }\|\boldsymbol{\Sigma}^{*}_{\mathbf{s}_{i}^{C}\mathbf{s}_{i}}{\left(\boldsymbol{\Sigma}^{*}_{\mathbf{s}_{i}\mathbf{s}_{i}}\right)}^{-1}\|_{\infty} \leq 1 - \alpha. $$

Here ***Σ*** ^∗^=(***Ω*** ^∗^)^−1^ and **s**_*i*_={*j*∈{1,…,*p*} | (***Ω*** ^∗^)_*ij*_≠0}. Assumption 2 under the multivariate normality guarantees that the support of ***Ω*** ^∗^ is recovered by SCIO with a high probability as the sample size gets large.

### Bipartite graphs inducing inconsistency with *ℓ*_1_ penalisation

Methods for sparse precision matrix estimation generally depend on an objective function (such as log-likelihood) and a penalty function or regulariser, which in a Bayesian setting is usually represented by the prior. The ideal penalty function for many problems would be the *ℓ*_0_ “norm” counting the number of non-zero elements: |*x*|_0_=*#*{*i*|*x*_*i*_≠0}. This *ℓ*_0_ function is not a proper norm, but it provides a very intuitive notion of sparsity. The main problem with its use is computational: using *ℓ*_0_-penalisation leads to very difficult non-convex combinatorial optimisation problems. The most common approach to avoid the computational challenges is to use *ℓ*_1_ penalisation as a convex relaxation of *ℓ*_0_. As mentioned above this works well in many cases but it comes with a price, since in addition to providing the sparsity, *ℓ*_1_ also regularises large non-zero values. Depending on the problem, as we demonstrate here, this effect can be substantial and may cause *ℓ*_1_-regularised methods to return totally meaningless results.

Intuitively, *ℓ*_1_-regularised methods are expected to fail when some elements of the true precision matrix become so large that their contribution to the penalty completely overwhelms the other parts of the objective and the penalty. One example where this happens is when some set of variables depends linearly on another set of variables. In such situation the covariance matrix can become ill-conditioned and the elements of its inverse, the precision matrix, grow. One example of when this happens is models with a linear latent variable structure.

**Fig. 1:**
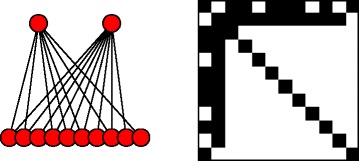
*Left*: Graphical representation of a latent variable model as an undirected graphical model for a case with somewhat sparse **A**. *Right*: The adjacency matrix of the graph showing the sparse pattern of non-zero elements in the corresponding precision matrix

Let us consider a model for $\mathbf {x} \in \mathbb {R}^{d_{1}}, \mathbf {y} \in \mathbb {R}^{d_{2}}$, where **y**=**A** **x**+*ε*. The graphical structure of the model and the corresponding precision matrix structure are illustrated in Fig. 1. Assuming $\mathbf {x} \sim \mathcal {N}(0, {\sigma _{x}^{2}} I), \epsilon \sim \mathcal {N}(0, \sigma _{\epsilon }^{2} I)$, the covariance of the concatenated vectors (**x** ^*T*^,**y** ^*T*^)^*T*^ is given by the block matrix

4 $$  \text{Cov}\left(\left(\mathbf{x}^{T}, \mathbf{y}^{T}\right)^{T}\right) = \mathbf{C} = {\sigma_{x}^{2}} \left(\begin{array}{lc} I & \mathbf{A}^{T} \\ \mathbf{A} & \mathbf{A} \mathbf{A}^{T} + \sigma_{\epsilon}^{2} I \end{array} \right).  $$

The covariance matrix has an analytic block matrix inverse [23]

5 $$ \mathbf{C}^{-1} = \sigma_{x}^{-2} \left(\begin{array}{cc} I + \sigma_{\epsilon}^{-2} \mathbf{A}^{T} \mathbf{A} & -\sigma_{\epsilon}^{-2} \mathbf{A}^{T} \\ -\sigma_{\epsilon}^{-2} \mathbf{A} & \sigma_{\epsilon}^{-2} I \end{array} \right).  $$

This precision matrix recapitulates the conditional independence result for Gaussian Markov random fields: the lower right block is diagonal because the variables in **y** are conditionally independent of each other given **x**. The matrix is clearly sparse, so we would intuitively assume sparse precision matrix estimation methods should be able to recover it. The non-zero elements do, however, depend on $\sigma _{\epsilon }^{-2}$ which can make them very large if the noise $\sigma _{\epsilon }^{2}$ is small.

It is possible to evaluate and bound the different terms of Eq. (1) evaluated at the ground truth for these models:

6 $$\begin{array}{*{20}l} \log\det(\mathbf{C}^{-1}) &= -(d_{1} + d_{2}) \log {\sigma_{x}^{2}} -d_{2} \log \sigma_{\epsilon}^{2} \end{array} $$

7 $$\begin{array}{*{20}l} -\text{trace}(\mathbf{C} \mathbf{C}^{-1}) &= -(d_{1} + d_{2}) \end{array} $$

8 $$\begin{array}{*{20}l} -\lambda |\mathbf{C}^{-1} |_{1} &< -\lambda \sigma_{x}^{-2} \sigma_{\epsilon}^{-2} (d_{2} + 2 | \mathbf{A} |_{1}). \end{array} $$

The magnitude of the penalty term (8) clearly grows very quickly as $\sigma _{\epsilon }^{2}$ decreases while the magnitudes of the two first log-likelihood terms (6) and (7) grow much more slowly as they only depend on $\log \sigma _{\epsilon }^{2}$. Thus the total value of Eq. (1) decreases without bound as $\sigma _{\epsilon }^{2}$ decreases.

Ignoring the ground truth, it is easy to see that one can construct an estimate **Ω** for which the objective remains bounded. If we assume |**C**|_*∞*_= max|*c*_*ij*_|≤1 (after normalisation), then

$$\text{trace}(\mathbf{C} \mathbf{\Omega}) \le | \mathbf{\Omega} |_{1}. $$

As the other terms only depend on **Ω** it is easy to choose **Ω** so that they remain bounded. The estimate **Ω** that yields these values will in many cases not have anything to do with **C** ^−1^, as seen in the experiments below.

Here Eq. (6) follows from the block matrix determinant identity [24]

$$ \det\left(\begin{array}{ll} \mathbf{A} & \mathbf{B} \\ \mathbf{C} & \mathbf{D} \end{array} \right) = \det(\mathbf{A} - \mathbf{B} \mathbf{D}^{-1} \mathbf{C}) \det(\mathbf{D}), $$

while Eq. (8) is based on a lower bound of the *ℓ*_1_ norm as the sum over all except the top-left block of the block matrix in Eq. (5).

## Results

### Synthetic example

We tested the performance of glasso, SCIO and CLIME as well as FMPL using the model structure corresponding to the bipartite graph introduced above. The performance of the methods was investigated by varying the noise variance $\sigma _{\epsilon }^{2}$, and the sample size *n*. The model matrix **A** was created as a (*d*_2_,*d*_1_)-array of independent normal random variables with mean 0 and variance 1. The majority of the tests were run using input dimensionality *d*_1_=2, output dimensionality *d*_2_=10 and noise variance $\sigma _{\epsilon }^{2} = 0.1^{2}$ but we also tested varying these settings. For each individual choice of noise and sample size, *k*=50 different matrices **A** were generated and the results were averaged.

Generating *n* samples using model described, data were normalised and analysed using the five different methods. We calibrated the methods in a way that number of edges in the resulting graph would match the true number. Similarly, we thresholded the naive method by taking inverse matrix directly to output the correct number of edges. The FMPL method has no direct tuning parameters so we used its OR mode results as such. Similar tuning is not possible in a real problem where the true number of edges is now known. The tuning represents the best possible results the methods could obtain with an oracle that provides an optimal regularisation parameter.

We evaluated the results using the Hamming distance between the ground truth and the inferred sparsity pattern, i.e. the number of incorrect edges and non-edges which were treated symmetrically. For methods returning the correct number of edges, this value is directly related to the precision *pr* through

$$d_{\text{Hamming}} = 2 (1 - pr) N_{\text{true positives}} $$

or conversely

$$pr = 1 - \frac{d_{\text{Hamming}}}{2 N_{\text{true positives}}}. $$

We will nevertheless use the Hamming distance as it enables fair comparison with FMPL that sometimes returns a different number of edges.

**Fig. 2:**
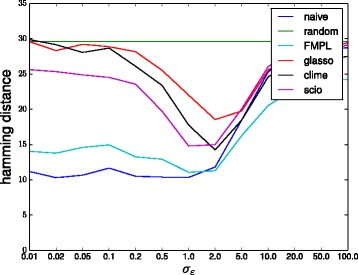
Performances of different methods on the bipartite graph model with 100 samples. (Lower values are better)

**Fig. 3:**
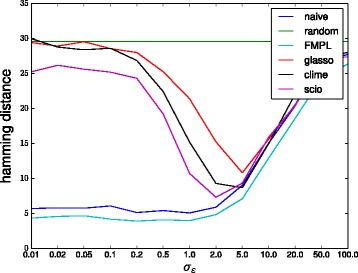
Performances of different methods on the bipartite graph model with 1000 samples. (Lower values are better)

Figures 2 and 3 show the Hamming distance obtained by the different methods as a function of the noise level when using 100 and 1000 samples, respectively. The results show that especially for low but also for high noise levels, the *ℓ*_1_-based methods all perform very poorly with especially glasso and CLIME performing very close to random guessing level for low noise levels *σ*_*ε*_≤0.1. The naive inverse and FMPL work much better up to moderate noise levels of *σ*_*ε*_≈2 after which the noise starts to dominate the signal and the performance of all methods starts to drop. SCIO is a little better than the other *ℓ*_1_-based methods but clearly worse than FMPL and naive in the low noise regime.

**Fig. 4:**
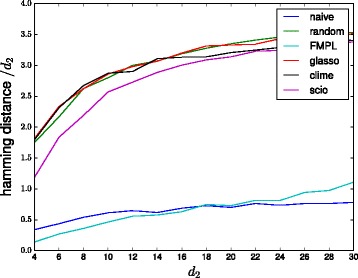
Performances of different methods on the bipartite graph model with varying output dimensionality. (Lower values are better)

Figure 4 shows the results when changing the output dimensionality *d*_2_ from 10. The results show that the performance of all *ℓ*_1_-based methods is very poor across all *d*_2_. Glasso performance is close to random guessing level across the entire range considered, while CLIME is slightly better for *d*_2_≥18 and SCIO slightly better across the entire range. Both FMPL and naive are significantly better than any of the *ℓ*_1_-based methods.

**Fig. 5:**
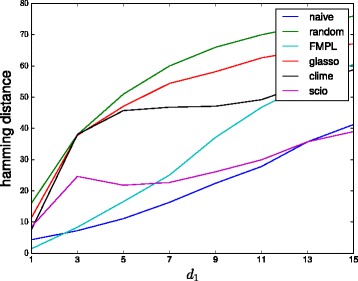
Performances of different methods on the bipartite graph model with varying input dimensionality. (Lower values are better)

Figure 5 shows the corresponding result when changing the input dimensionality *d*_1_. The results are now quite different as all methods are better than random especially for larger values. SCIO still outperforms CLIME which outperforms glasso. FMPL is really accurate for small *d*_1_ but degrades for larger *d*_1_ while the naive method is the most accurate in almost all cases.

**Fig. 6:**
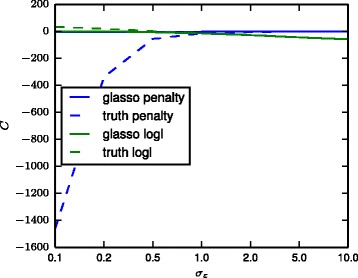
Contributions of the different terms of the glasso objective (1) for the bipartite graph model with 1000 samples. The *green curves* show the contributions of the first two terms of Eq. (1) and the *blue curves* show the contributions of the last penalty term. *Solid lines* show the result of the glasso optimal solution while *dashed lines* show the result for the true solution

To further illustrate the behaviour of glasso on these examples, Fig. 6 shows the contributions of the different parts of the glasso objective function (1) as a function of the noise level both for the true solution (“truth”) as well as the glasso solution. The results show that for low noise levels the penalty incurred by the true solution becomes massive. The glasso solution has a much lower log-likelihood (“logl”) than ground truth but this is amply compensated by the significantly smaller penalty. As the noise increases, the penalty of the true solution decreases and the glasso solution converges to similar values.

### Necessity of assumption 1

**Fig. 7:**
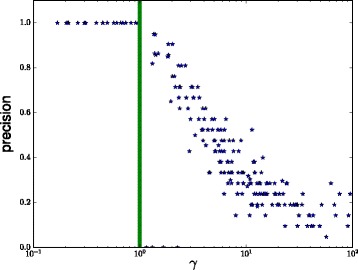
Precision of glasso on infinite data as a function of the norm *γ* of Assumption 1 and Eq. (3). Values to the *left* of the *green vertical line* satisfy this condition while values to the *right* violate it. (Higher values are better)

It can be checked that the norm *γ* in Assumption 1 and Eq. (3) for bipartite graph models presented above depends on the scale of **A**. We took advantage of this by creating examples with different values of *γ* and testing the precision of glasso using the true covariance which corresponds to infinite data limit. The results of this experiment are shown in Fig. 7. The results verify that glasso consistently yields perfect results when *γ*<1 which is a part of the sufficient conditions for consistency of glasso. As *γ* grows and the sufficient conditions are no longer satisfied, it is clearly seen that the accuracy of glasso starts to deteriorate rapidly. This suggests that the sufficient condition of Assumption 1 is in practice also necessary to ensure consistence.

### Inconsistency for models of real gene expression data

We tested how often the problems presented above appear in real data using the “TCGA breast invasive carcinoma (BRCA) gene expression by RNAseq (IlluminaHiSeq)” data set [25] downloaded from https://genome-cancer.ucsc.edu/proj/site/hgHeatmap/. The data set contains gene expression measurements for 20530 genes for *n*=1215 samples. After removing genes with a constant expression across all samples there are *p*=20252 genes remaining.

In order to test the methods we randomly sampled subsets of *d* genes and considered the correlation matrix **C**_0_ over that subset. We generated sparse models with known ground truth by computing the corresponding precision matrix ***Λ***_0_ from the empirical correlation matrix, setting elements with absolute values below chosen cutoff *δ*=0.1 to 0 to obtain

9 $$  \boldsymbol{\Lambda}_{ij} =\left\{ \begin{array}{ll} (\boldsymbol{\Lambda}_{0})_{ij} \quad &\text{if}~ |(\boldsymbol{\Lambda}_{0})_{ij}| > \delta \\ 0 \quad &\text{otherwise} \end{array} \right.  $$

and the testing covariance matrix **C**=***Λ*** ^−1^. The cutoff lead to networks that were sparse with on average 60 % zeros in the precision matrix.

**Fig. 8:**
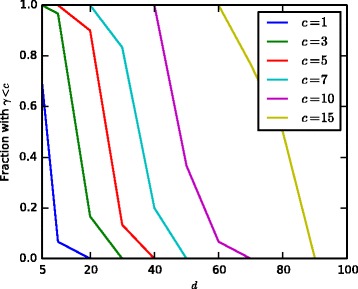
Testing the condition of Assumption 1 of [22] in Eq. (3) on real gene expression data showing the fraction of random subsets of *d* genes that fulfil the requirement and various relaxations. The condition (3) requires *γ*<1, but the figure shows results also for larger *γ* cutoffs, denoted by *c*

Figure 8 shows the fraction of covariances derived from random subsets of *d* genes that satisfy the Assumption 1 of [22] (*c*=1) as well as the fraction of values below more relaxed bounds. The figure shows that the assumption is reliably satisfied only for very small *d* while for *d*≥20, the assumption is essentially never satisfied. Based on the results of Fig. 7 it is likely that glasso results will degrade significantly for *γ*>10 and beyond which are very common for large networks.

We further studied how accurately glasso can recover the graphical structures when the data were generated using the precision matrices described above. We used a similar thresholding with a cut-off value of 0.1 in order to first form sparse precision matrices for a random subset of genes with given dimension. These matrices were then inverted to obtain covariance matrices. We checked that the resulting matrices were positive definite and then used them to sample multivariate normal data with zero mean with different sample sizes.

**Fig. 9:**
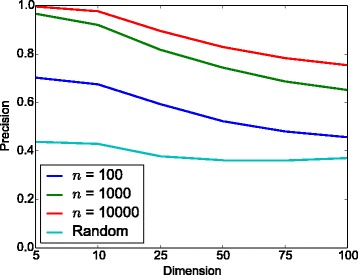
Average precisions for glasso with different dimensions and sample sizes of the real gene expression data, higher values are better. In these experiments, 50 data sets were created. We encountered convergence problems with few of the data sets and the corresponding results were omitted when computing the average values shown here. The precision obtained by random guessing is also illustrated

The obtained data sets were centred and scaled before computing the sample covariance which was used as input to the glasso algorithm. The regularisation parameter was chosen with the aid of the ground truth graph, so that the the graph identified by glasso would contain as many edges as there were in the real graph. Results are shown in Fig. 9. The results show that glasso performance decreases as the network size increases and is approaching that of random guessing for the largest networks considered here.

**Fig. 10:**
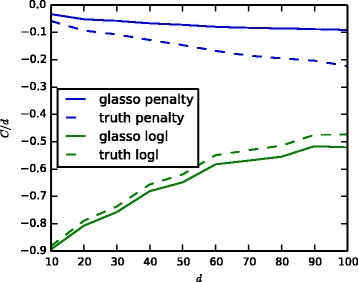
Average contributions of the different terms of the glasso objective function (1) on real gene expression data over random subsets of *d* genes. The values are shown for the *ℓ*_1_ penalty term as well as the unnormalised log-likelihood, divided by *d* to make them comparable. *Solid lines* show the values for glasso result while *dashed lines* show the result for ground truth

Figure 10 shows the contributions of different parts of the glasso objective function (1) as a function of the number of genes *d*. The regularisation parameter *λ* of glasso was tuned to return a solution with the same number of edges as in the true solution. We used the glasso implementation of scikit-learn [26], which ignores the diagonal terms of ***Ω*** when computing the penalty. The figure shows clearly how the penalty term for the true solution increases superlinearly as a function of *d*. (A linear increase would correspond to a horizontal line.) The result is even more striking given that the optimal *λ* decreases slightly as *d* increases. The penalty contribution for glasso solution increases much more slowly. The excess loss in log-likelihood from glasso solution increases as *d* increases, but this is compensated by a larger saving in the penalty. Together these suggest that glasso solutions are likely to remain further away from ground truth as *d* increases.

## Discussion

The class of models with bipartite graphs presented above is an interesting example of models that have a very clear sparse structure, which all *ℓ*_1_-penalisation-based methods seem unable to recover even in the limit of infinite data. This class complements the previously considered examples of models where glasso is inconsistent including the “two neighbouring triangles” model of [27] and the star graph of [22], the latter of which can be seen as a simple special case of our example.

An important question arising from our investigation is how significant the discovered limitation to inferring sparse covariance matrices is in practice, i.e. how common are the (nearly) bipartite structures in real data sets. Given the popularity and success of linear models in diverse applications it seems plausible such structures could often exist in real data sets, either as an intrinsic property or as a result of some human intervention, e.g. through inclusion of partly redundant variables.

The gene expression data set is a natural example of an application where graphical model structure learning has been considered. The original glasso paper [7] contained an example on learning gene networks, although from proteomics data. Other authors (e.g. [28]) have applied Gaussian graphical models and even glasso (e.g. [29]) to gene network inference from expression data. Our experiments on the TCGA gene expression data suggest that in such applications it is advisable to consider the conditions for the consistency of *ℓ*_1_-penalised methods very carefully when planning to apply those.

Previous publications presenting new methods for sparse precision matrix have typically tested the method on synthetic examples where the true precision matrix is specified to contain mostly small values. Specifying the precision matrix provides a convenient way to generate test cases as the sparsity pattern can be defined very naturally through it. At the same time, this excludes any models that have an ill-conditioned covariance. As shown by our example, such ill-conditioned covariances arise very naturally from model structures that are plausible from the application perspective. The example presented in this paper thus represents a very useful additional test case for method developers and benchmarkers.

## Conclusions

Our results strongly suggest that users of the numerous *ℓ*_1_-penalised and other *ℓ*_1_-based sparse precision matrix and Gaussian graphical model structure learning methods should be very careful about checking whether the conditions of consistency for precision matrix estimation are likely to be fulfilled in the application area of interest. The consistency conditions are typically presented in a form which requires knowing the ground truth which makes it difficult to test them directly. Developing alternative criteria that can be checked more easily in practice would be an important avenue of future research for these methods.

## References

[1] Holter NS, Mitra M, Maritan A, Cieplak M, Banavar JR, Fedoroff NV (2000). Fundamental patterns underlying gene expression profiles: simplicity from complexity. Proc Natl Acad Sci U S A.

[2] Liao JC, Boscolo R, Yang YL, Tran LM, Sabatti C, Roychowdhury VP (2003). Network component analysis: reconstruction of regulatory signals in biological systems. Proc Natl Acad Sci U S A.

[3] Sabatti C, James GM (2006). Bayesian sparse hidden components analysis for transcription regulation networks. Bioinformatics.

[4] Sanguinetti G, Lawrence ND, Rattray M (2006). Probabilistic inference of transcription factor concentrations and gene-specific regulatory activities. Bioinformatics.

[5] Arrieta-Ortiz ML, Hafemeister C, Bate AR, Chu T, Greenfield A, Shuster B, Barry SN, Gallitto M, Liu B, Kacmarczyk T, Santoriello F, Chen J, Rodrigues CDA, Sato T, Rudner DZ, Driks A, Bonneau R, Eichenberger P (2015). An experimentally supported model of the Bacillus subtilis global transcriptional regulatory network. Mol Syst Biol.

[6] Meinshausen N, Bühlmann P (2006). High-dimensional graphs and variable selection with the lasso. Ann Stat.

[7] Friedman J, Hastie T, Tibshirani R (2008). Sparse inverse covariance estimation with the graphical lasso. Biostatistics.

[8] Cai T, Liu W, Luo X (2011). A constrained *ℓ*_1_ minimization approach to sparse precision matrix estimation. J Am Stat Assoc.

[9] Zhao P, Yu B (2006). On model selection consistency of lasso. J Mach Learn Res.

[10] Whittaker J (1990). Graphical Models in Applied Multivariate Statistics.

[11] Lauritzen SL (1996). Graphical Models.

[12] Tibshirani R (1996). Regression shrinkage and selection via the lasso. J R Stat Soc Ser B.

[13] Yuan M, Lin Y (2007). Model selection and estimation in the Gaussian graphical model. Biometrika.

[14] Banerjee O, El Ghaoui L, d’Aspremont A (2008). Model selection through sparse maximum likelihood estimation for multivariate Gaussian or binary data. J Mach Learn Res.

[15] Peng J, Wang P, Zhou N, Zhu J (2009). Partial correlation estimation by joint sparse regression models. J Am Stat Assoc.

[16] Hsieh C, Sustik MA, Dhillon IS, Ravikumar PD (2014). QUIC: quadratic approximation for sparse inverse covariance estimation. J Mach Learn Res.

[17] Liu W, Luo X (2015). Fast and adaptive sparse precision matrix estimation in high dimensions. J Multivar Anal.

[18] Leppä-aho J, Pensar J, Roos T, Corander J. Learning Gaussian graphical models with fractional marginal pseudo-likelihood. arXiv:1602.07863 [stat.ML]. 2016.

[19] Geiger D, Heckerman D (2002). Parameter priors for directed acyclic graphical models and the characterization of several probability distributions. Ann Stat.

[20] Consonni G, Rocca LL (2012). Objective Bayes factors for Gaussian directed acyclic graphical models. Scand J Stat.

[21] Pensar J, Nyman H, Niiranen J, Corander J. Marginal pseudo-likelihood learning of discrete Markov network structures. Bayesian Anal. doi:10.1214/16-BA1032.

[22] Ravikumar P, Wainwright MJ, Raskutti G, Yu B (2011). High-dimensional covariance estimation by minimizing *ℓ*_1_-penalized log-determinant divergence. Electron J Stat.

[23] Lu TT, Shiou SH (2002). Inverses of 2×2 block matrices. Comput Math Appl.

[24] Powell PD. Calculating determinants of block matrices. 2011. arXiv:1112.4379 [math.RA].

[25] Cancer Genome Atlas Network (2012). Comprehensive molecular portraits of human breast tumours. Nature.

[26] Pedregosa F, Varoquaux G, Gramfort A, Michel V, Thirion B, Grisel O, Blondel M, Prettenhofer P, Weiss R, Dubourg V, Vanderplas J, Passos A, Cournapeau D, Brucher M, Perrot M, Duchesnay E (2011). Scikit-learn: Machine learning in Python. J Mach Learn Res.

[27] Meinshausen N (2008). A note on the Lasso for Gaussian graphical model selection. Stat Probab Lett.

[28] Ma S, Gong Q, Bohnert HJ (2007). An Arabidopsis gene network based on the graphical Gaussian model. Genome Res.

[29] Menéndez P, Kourmpetis YAI, ter Braak CJF, van Eeuwijk FA (2010). Gene regulatory networks from multifactorial perturbations using Graphical Lasso: application to the DREAM4 challenge. PLoS One.

